# PRO: Carbapenems should be used for ALL infections caused by ceftriaxone-resistant Enterobacterales

**DOI:** 10.1093/jacamr/dlab013

**Published:** 2021-02-24

**Authors:** David L Paterson, Burcu Isler, Patrick N A Harris

**Affiliations:** 1 University of Queensland Centre for Clinical Research (UQCCR), RBWH Campus, Brisbane, Australia; 2 Infectious Diseases Unit, Royal Brisbane and Women’s Hospital, Brisbane, Australia; 3 Herston Infectious Diseases Institute (HeIDI), Brisbane, Australia; 4 Central Microbiology Laboratory, Pathology Queensland, Brisbane, Australia

## Abstract

Ceftriaxone resistance in the Enterobacterales is typically the result of production of ESBLs or AmpC β-lactamases. The genes encoding these enzymes are often co-located with other antibiotic resistance genes leading to resistance to aminoglycosides, quinolones and trimethoprim/sulfamethoxazole. Carbapenems are stable to ESBLs and AmpC giving them reliable *in vitro* activity against producers of these β-lactamases. In contrast, piperacillin/tazobactam and amoxicillin/clavulanate are compromised by co-production of OXA-1, which is not inhibited by tazobactam or clavulanate. These *in vitro* findings provide an explanation for the MERINO trial outcomes, where 3.7% (7/191) randomized to meropenem died compared with 12.3% (23/187) randomized to piperacillin/tazobactam as definitive treatment of bloodstream infection due to ceftriaxone-resistant organisms. No randomized trials have yet put cefepime and carbapenems head to head, but some observational studies have shown worse outcomes with cefepime. We argue that carbapenems are the antibiotics of choice for ceftriaxone-resistant Enterobacterales.

ESBL-producing Enterobacterales are priority 1 (‘critical’) on the WHO priority pathogens list for research and development of new antibiotics.^1^ However, at the present time, carbapenems remain the treatment of choice for infections due to ceftriaxone-resistant Enterobacterales. First, we believe this to be the case because the only randomized controlled trial comparing options for ceftriaxone-resistant organisms (the MERINO trial) showed a lower all-cause mortality when carbapenems were used. Secondly, we believe that ESBLs themselves compromise alternatives like cefepime or co-produced enzymes (OXA-1) that are not inhibited by tazobactam or clavulanate. The stability of carbapenems to ESBLs and AmpC β-lactamases gives this antibiotic class a natural advantage over comparators. The purpose of this paper is to provide the evidence for this position.

## Bloodstream infection

The MERINO trial (NCT02176122/ACTRN12613000532707) was the first multi-country randomized clinical trial (RCT) evaluating treatment options for patients with ceftriaxone-resistant *Escherichia coli* or *Klebsiella pneumoniae* bloodstream infection.^2^ The study compared piperacillin/tazobactam and meropenem as definitive antibiotic treatment, once ceftriaxone resistance and piperacillin/tazobactam and meropenem susceptibility were confirmed. All-cause, 30 day mortality was chosen as the primary endpoint since in many cases the cause of death is highly subjective or multifactorial. The study found that 12.3% (23 of 187) patients randomized to piperacillin/tazobactam died within 30 days of randomization compared with 3.7% (7 of 191) randomized to meropenem. While acknowledging that some deaths were likely unrelated to the infection or its treatment, the trial data support the primary place of carbapenems for treatment of bloodstream infection.

Criticism of the MERINO trial has included commentary that imbalance in baseline characteristics may have been responsible for the trial findings.^3^ We argue that while some baseline characteristics favouring poor outcome were more common in the piperacillin/tazobactam group, and likely did lead to the demise of some patients, meropenem treated patients had higher APACHE II scores and had a longer time until administration of appropriate antibiotics. The MERINO trial showed that 138/185 (74.6%) of all those who received meropenem had clinical and microbiological success (defined as survival, resolution of fever, normalized white cell count and negative blood cultures) at 4 days from receipt of the study drug as did 112/154 (72.7%) of those who received piperacillin/tazobactam and survived. In stark contrast, only 9/23 (39.1%) of those who received piperacillin/tazobactam and died had clinical and microbiological success. It appears that most deaths were, in fact, attributable to failure of piperacillin/tazobactam to resolve the infection. In some patients, suboptimal treatment of these bloodstream infections due to ESBL producers may ‘push’ patients with co-morbidities ‘over the edge’, resulting in premature death, even if infection cannot clearly be regarded as the proximal cause of death.

Microbiologically, there is rationale for the MERINO trial findings. Isolates co-harbouring ESBL genes (especially *bla*_CTX-M-15_) and the OXA-1 gene were associated with elevated piperacillin/tazobactam MICs and an increase in 30 day mortality.^4^ OXA-1 is a β-lactamase that is relatively resistant to inhibition by β-lactamases such as tazobactam. The CTX-M-15/OXA-1 β-lactamase combination was geographically distributed amongst all regions in which patients were enrolled in the MERINO trial: Middle East (45%), Turkey/Mediterranean Europe region (43%), South Africa (38%) and Singapore (33%), Australia, New Zealand and Canada combined (21%).^4^ Unfortunately there is no easy way in which clinical microbiology laboratories can detect the presence of OXA-1.

An editorial accompanying the publication of the MERINO trial concluded that the search for a carbapenem-sparing option for bloodstream infection due to ESBL producers continues.^5^ Could cefepime be one such option? Cefepime is a substrate for ESBLs and ESBL production often causes MIC elevation. Previous cefepime susceptibility breakpoints used in Europe and the USA were likely too high and resulted in categorization of many ESBL producers as susceptible.^6^ This was associated with treatment failures for patients treated with cefepime. Cefepime susceptibility breakpoints were subsequently lowered (to ≤1 mg/L and ≤2 mg/L, by EUCAST and CLSI, respectively) to leave most ESBL producers outside the susceptible range.^7^^,^^8^ However, in a propensity matched series of patients with bloodstream infection due to ESBL producers with *in vitro* cefepime susceptibility, 14 day mortality in cefepime-treated patients was 2.87 times higher than carbapenem-treated patients.^9^ One explanation for this is lower cefepime efficacy in the presence of higher bacterial inoculum as demonstrated *in vitro* and in animal studies.^10^^,^^11^ Another explanation is inadequately dosed cefepime failing to achieve therapeutic targets. There are no controlled clinical studies testing these hypotheses.

We have recently initiated an RCT (MERINO-3) comparing meropenem with ceftolozane/tazobactam for bloodstream infections due to ceftriaxone-resistant Enterobacterales (NCT04238390).^12^ The PETERPEN trial (NCT03671967) compares meropenem with piperacillin/tazobactam for more than 1000 patients with ceftriaxone-resistant *E. coli* or *K. pneumoniae* bloodstream infection.^13^ Planned exclusion criteria make the PETERPEN study population of lower severity of illness than the MERINO trial population. Until results of these trials are available, we maintain that carbapenems should continue to be used for bloodstream infections due to ceftriaxone-resistant Enterobacterales.

## Ventilator-associated pneumonia and complicated intra-abdominal infection

By extension of the results seen with bloodstream infection, piperacillin/tazobactam would not be an appropriate choice for ventilator-associated pneumonia or complicated intra-abdominal infection due to ESBL- or AmpC-producing organisms. In an evaluation of patients with ESBL producers in a pneumonia trial, there were 4/13 (31%) cefepime-treated patients with clinical failure, whereas all 10 patients had clinical success in the imipenem arm.^14^ Some new antibiotics, such as ceftazidime/avibactam, cefiderocol and ceftolozane/tazobactam have been compared with carbapenems for treatment of hospital-acquired pneumonia or complicated intra-abdominal infection. None of these RCTs has shown that an alternative to carbapenems has resulted in superior outcomes.^15–19^

We conclude that carbapenems should continue to be used for all patients with ventilator-associated pneumonia or complicated intra-abdominal infection due to ceftriaxone-resistant Enterobacterales.

## Complicated urinary tract infections

In the MERINO trial, 6.9% (7/102) piperacillin/tazobactam treated patients with a urinary tract source of bacteraemia died as compared with 3.1% (4/128) randomized to meropenem. In a UTI trial, two of six patients in the cefepime arm had clinical failure when infected with an ESBL producer, despite cefepime MICs being ≤2 mg/L.^20^ Clinical failure in these cases represented progression to septic shock. In contrast, clinical failure occurred in only 1 of 33 randomized to ertapenem.^20^

Ceftriaxone-resistant Enterobacterales are frequently MDR so that urinary tract isolates are typically resistant to fluoroquinolones and trimethoprim/sulfamethoxazole. No RCT has compared these options, when susceptible, with carbapenems for treatment of UTI. Other orally administered alternatives are also compromised. Amoxicillin/clavulanate suffers the same as piperacillin/tazobactam when organisms co-produce ESBLs and the OXA-1 β-lactamase. Nitrofurantoin and orally administered fosfomycin have utility limited only to the lower urinary tract. The FOREST trial (NCT02142751) has compared fosfomycin with meropenem for treatment of *E. coli* bloodstream infection of urinary tract origin.^21^ Oral therapy is permitted from the fifth day of therapy if clinical improvement is achieved. This comprises oral fosfomycin trometamol in the fosfomycin arm and one of ciprofloxacin, amoxicillin/clavulanate or trimethoprim/sulfamethoxazole in the meropenem arm. The findings of the study are awaited with interest to determine if a fosfomycin-based regimen is equivalent to the carbapenem backbone.

A new, orally administered carbapenem antibiotic, tebipenem, has recently been evaluated in a large RCT evaluating therapies for complicated urinary tract infection. This new carbapenem may present a new treatment option for urinary tract infection due to ESBL- or AmpC-producing bacteria.

## Conclusions

Carbapenems are stable to the effects of common β-lactamases, except for carbapenemases. ESBLs may compromise the activity of cefepime and co-produced OXA-1 reduces the effectiveness of β-lactamase inhibitors like tazobactam. As was shown in the MERINO trial, the clinical effectiveness of carbapenems has never been surpassed in treatment of ceftriaxone-resistant Enterobacterales (Table 1). A variety of new antibiotics outside the carbapenem class are now available for treatment of carbapenem-resistant Enterobacterales and should be reserved for these infections until further trial data becomes available.

**Table 1.** Disadvantages of alternative therapies for ceftriaxone-resistant Enterobacterales

Carbapenem-sparing alternative therapies for ESBL and AmpC producers	Disadvantages
Piperacillin/tazobactam	Failed to demonstrate non-inferiority against meropenem in an RCT of patients with bloodstream infections. Efficacy frequently compromised by presence of OXA-1 co-production. Automated systems may provide unreliable susceptibility results for piperacillin/tazobactam in ESBL producers (especially with OXA-1 co-production).
Cefepime	May be hydrolysed by some ESBLs. Propensity matched observational study showed higher mortality with cefepime than carbapenems, even when cefepime susceptibility was demonstrated.
Ceftazidime/avibactam, ceftolozane/ tazobactam, cefiderocol	For bloodstream infections, comparable or superior efficacy to carbapenems has not yet been demonstrated in RCTs. More expensive than generic carbapenems or not yet widely available. Should be reserved for organisms where few alternatives exist (e.g. KPC producers, carbapenem-resistant non-fermenters).
Fosfomycin	Efficacy of oral formulation limited to uncomplicated lower urinary tract infections. IV formulation not widely available.

## Transparency declarations

D.L.P. has received research grants from Merck, Pfizer and Shionogi outside of the submitted work. He has also received personal fees from Merck, Pfizer, Shionogi, Shionogi, Lysovant, The Medicines Company, Entasis, Venatorx, BioMérieux and Accelerate. P.N.A.H. has received research grants from Merck Sharp and Dohme (MSD), Sandoz and Shionogi Ltd, outside of the submitted work, as well as personal fees from Pfizer and Sandoz. B.I. has none to declare.

## References

[1] WHO. Prioritization of Pathogens to Guide Discovery, Research and Development of New Antibiotics for Drug-Resistant Bacterial Infections, Including Tuberculosis. 2017. https://www.who.int/medicines/areas/rational_use/prioritization-of-pathogens/en/.

[2] Harris PN , Tambyah PA, Lye DC et al Effect of piperacillin-tazobactam vs meropenem on 30-day mortality for patients with E coli or *Klebsiella pneumoniae* bloodstream infection and ceftriaxone resistance: a randomized clinical trial. JAMA 2018; 320: 984–94. 30208454 10.1001/jama.2018.12163 PMC6143100

[3] Rodríguez-Baño J , Gutiérrez-Gutiérrez B, Kahlmeter G. Antibiotics for ceftriaxone-resistant Gram-negative bacterial bloodstream infections. JAMA 2019; 321: 612–3. 10.1001/jama.2018.19345 30747960

[4] Henderson A , Paterson DL, Chatfield M et al Association between minimum inhibitory concentration, β-lactamase genes and mortality for patients treated with piperacillin/tazobactam or meropenem from the MERINO study. Clin Infect Dis 2020; doi: 10.1093/cid/ciaa1479. 10.1093/cid/ciaa1479 33106863

[5] Hayden MK , Won SY. Carbapenem-sparing therapy for extended-spectrum β-lactamase–producing E coli and *Klebsiella pneumoniae* bloodstream infection: the search continues. JAMA 2018; 320: 979–81. 30208439 10.1001/jama.2018.12565

[6] Paterson DL , Ko W-C, Von Gottberg A et al Outcome of cephalosporin treatment for serious infections due to apparently susceptible organisms producing extended-spectrum β-lactamases: implications for the clinical microbiology laboratory. J Clin Microbiol 2001; 39: 2206–12. 11376058 10.1128/JCM.39.6.2206-2212.2001 PMC88112

[7] Kahlmeter G. Breakpoints for intravenously used cephalosporins in Enterobacteriaceae–EUCAST and CLSI breakpoints. Clin Microbiol Infect 2008; 14 Suppl 1: 169–74. 18154542 10.1111/j.1469-0691.2007.01856.x

[8] CLSI. *Performance Standards for Antimicrobial Susceptibility Testing—Twenty-Sixth Informational Supplement: M100-S26*. 2016.

[9] Wang R , Cosgrove SE, Tschudin-Sutter S et al Cefepime therapy for cefepime-susceptible extended-spectrum β-lactamase-producing Enterobacteriaceae bacteremia. Open Forum Infect Dis 2016; 3: ofw132. 27419191 10.1093/ofid/ofw132 PMC4942761

[10] Reese AM , Frei CR, Burgess DS. Pharmacodynamics of intermittent and continuous infusion piperacillin/tazobactam and cefepime against extended-spectrum β-lactamase-producing organisms. Int J Antimicrob Agents 2005; 26: 114–9. 16029947 10.1016/j.ijantimicag.2005.06.004

[11] Thomson KS , Moland ES. Cefepime, piperacillin-tazobactam, and the inoculum effect in tests with extended-spectrum β-lactamase-producing Enterobacteriaceae. Antimicrob Agents Chemother 2001; 45: 3548–54. 11709338 10.1128/AAC.45.12.3548-3554.2001 PMC90867

[12] Ceftolozane-Tazobactam Versus Meropenem for ESBL and AmpC-Producing Enterobacterales Bloodstream Infection (MERINO III). https://clinicaltrials.gov/ct2/show/NCT04238390. 10.1186/s13063-021-05206-8 PMC8060904 33888139

[13] PipEracillin Tazobactam Versus mERoPENem for Treatment of Bloodstream Infections Caused by Cephalosporin-Resistant Enterobacteriaceae (PETERPEN). https://clinicaltrials.gov/ct2/show/NCT03671967. 10.1136/bmjopen-2020-040210 PMC7871690 33558347

[14] Zanetti G , Bally F, Greub G et al Cefepime versus imipenem-cilastatin for treatment of nosocomial pneumonia in intensive care unit patients: a multicenter, evaluator-blind, prospective, randomized study. Antimicrob Agents Chemother 2003; 47: 3442–7. 14576100 10.1128/AAC.47.11.3442-3447.2003 PMC253800

[15] Torres A , Zhong N, Pachl J et al Ceftazidime-avibactam versus meropenem in nosocomial pneumonia, including ventilator-associated pneumonia (REPROVE): a randomised, double-blind, phase 3 non-inferiority trial. Lancet Infect Dis 2018; 18: 285–95. 29254862 10.1016/S1473-3099(17)30747-8

[16] Kollef MH , Nováček M, Kivistik Ü et al Ceftolozane–tazobactam versus meropenem for treatment of nosocomial pneumonia (ASPECT-NP): a randomised, controlled, double-blind, phase 3, non-inferiority trial. Lancet Infect Dis 2019; 19: 1299–311. 31563344 10.1016/S1473-3099(19)30403-7

[17] Wunderink R , Matsunaga Y, Ariyasu M et al Cefiderocol versus high-dose, extended-infusion meropenem for the treatment of Gram-negative nosocomial pneumonia (APEKS-NP): a randomised, double-blind, phase 3, non-inferiority trial. Lancet Infect Dis 2020; doi: 10.1016/S1473-3099(20)30731-3. 10.1016/S1473-3099(20)30731-3 33058798

[18] Mendes RE , Castanheira M, Woosley LN et al Molecular β-lactamase characterization of aerobic Gram-negative pathogens recovered from patients enrolled in the ceftazidime-avibactam phase 3 trials for complicated intra-abdominal infections, with efficacies analyzed against susceptible and resistant subsets. Antimicrob Agents Chemother 2017; 61: e02447-16. 28348155 10.1128/AAC.02447-16 PMC5444185

[19] Popejoy MW , Paterson DL, Cloutier D et al Efficacy of ceftolozane/tazobactam against urinary tract and intra-abdominal infections caused by ESBL-producing *Escherichia coli* and *Klebsiella pneumoniae*: a pooled analysis of Phase 3 clinical trials. J Antimicrob Chemother 2017; 72: 268–72. 27707990 10.1093/jac/dkw374

[20] Seo YB , Lee J, Kim YK et al Randomized controlled trial of piperacillin-tazobactam, cefepime and ertapenem for the treatment of urinary tract infection caused by extended-spectrum β-lactamase-producing *Escherichia coli*. BMC Infect Dis 2017; 17: 404. 28592240 10.1186/s12879-017-2502-x PMC5463388

[21] Rosso-Fernández C , Sojo-Dorado J, Barriga A et al Fosfomycin versus meropenem in bacteraemic urinary tract infections caused by extended-spectrum β-lactamase-producing *Escherichia coli* (FOREST): study protocol for an investigator-driven randomised controlled trial. BMJ Open 2015; 5: e007363. 10.1136/bmjopen-2014-007363 PMC4386243 25829373

